# Label-Free Quantification of Anti-TNF-α in Patients Treated with Adalimumab Using an Optical Biosensor

**DOI:** 10.3390/s18030691

**Published:** 2018-02-26

**Authors:** Rosa Helena Bustos, Carlos Zapata, Efraín Esteban, Julio-César García, Edwin Jáuregui, Diego Jaimes

**Affiliations:** 1 Evidence-Based Therapeutics Group, Clinical Pharmacology, Universidad de La Sabana, 140013 Chía, Colombia; carloszaam@unisabana.edu.co (C.Z.); efrainesma@unisabana.edu.co (E.E.); Julio.Garcia@unisabana.edu.co (J.-C.G.); diegojf@unisabana.edu.co (D.J.); 2 Riesgo de Fractura S.A.-CAYRE, 110221 Bogotá, Colombia; apoyomedico@cayre.co

**Keywords:** biological drug, therapeutic drug monitoring (TDM), optical biosensor

## Abstract

This study describes the development of an immunosensory label-free quantification methodology based on surface plasmon resonance (SPR) and its applicability in measuring/evaluating therapeutic drug monitoring (TDM) of anti-TNF-α monoclonal antibody (adalimumab) in rheumatoid arthritis (RA) patients. The experimental parameters evaluated in this study were immobilising ligands by pre-concentration assays, sensor surface regeneration, ascertaining the method’s sensitivity and correlating the results from quantifying plasma samples by ELISA immunoassay. The results showed that TNF-α quantification values (in RU) were significantly different when comparing patients (~50–250 RU) to controls (~10–20 RU). Likewise, there was 0.97 correlation for patients and 0.91 for healthy volunteers using SPR and ELISA comparison methodologies. SPR immunosensory detection provided a precise, sensitive strategy, along with real-time determination, for quantifying adalimumab, having great potential for clinical routine regarding TDM.

## 1. Introduction

Anti-tumour necrosis factor (TNF) monoclonal antibodies (mAb) are prescribed for patients suffering chronic diseases, such as rheumatoid arthritis (RA), ankylosing spondylitis (AS), psoriatic arthritis (PsA), and intestinal bowel diseases (IBD), such as ulcerative colitis (UC) and Crohn’s disease (CD), in both adults and children [1]. Such drugs include infliximab (Remicade), a mouse-human IgG1-kappa anti-TNF-α mAb, etanercept (Enbrel), a human TNF receptor 2 and IgG1 Fc fusion protein and adalimumab (Humira), a human IgG1-kappa anti-TNF-α complement mAb. TNF-α inhibitors can dramatically reduce autoimmune disease activity and induce remission in some patients, although not all patients respond appropriately/as expected [2].

The mechanisms which could explain such therapeutic failure are still not clear and do not receive necessary attention on many occasions. Amongst the requirements stipulated by regulatory agencies, such as the US Food and Drugs Administration (FDA), European Medicines Agency (EMA) and the World Health Organisation (WHO), therapeutic drug monitoring (TDM) is necessary for minimising the risks associated with therapeutic failure and optimising therapeutic effects [3,4,5,6], thereby rationalising treatment costs [7,8,9,10]. Enormous variations in biologics’ plasma levels cannot be explained by classic pharmacokinetic (PK) parameters governing chemically-synthesised drugs [11,12,13,14,15,16]. Biopharmaceutical products represent a great challenge for treating doctors, given the particular behaviour of these molecules which have been seen to be associated with forming anti-drug antibodies (ADA) [17,18,19] and a PK/pharmacodynamic (PD) profile having significant inter-individual variation [20,21,22]. This leads to problems regarding overdosing, therapeutic failure [23,24] and drug interactions [25,26,27]; this increases adverse reaction frequency [28,29,30,31] (some potentially fatal) [32,33,34,35] which can lead to patients abandoning their treatment [36,37].

Measuring/monitoring these drugs is often expensive, involving prolonged times and (for some molecules) sensitivity and specificity levels lead to questioning its usefulness in both clinical practice and the research field [38]. TDM for adalimumab plasma levels has been widely established over the past few years. The methodologies most commonly used for measuring/monitoring adalimumab have included ELISA [39,40], electrochemiluminescence [41], radio-immune assays [42,43,44] and solid-phase protein adsorption [42]. Surface plasmon resonance (SPR) assay is another analysis format for biologics since its use has been established for determining antigen-antibody interaction. The assay for determining anti-TNF-α plasma levels in this study comes from a methodology referred to in some regulations [3] and pharmacopeia [45] for evaluating antibody or biologics using SPR. This methodology’s use has become more widespread recently due to shorter analysis times and results being available in real-time [46]. SPR can be used for assays with human antibodies, or antibody Fc region constructs, as they enable diluted serum or plasma samples to be analysed and bound to a protein drug’s independently immobilised domains or for simultaneously determining crossed reactivity between drugs and homologues, such as endogenous proteins; this facilitates determining antibody response specificity [47].

SPR nanobiosensors belong to a group of optical biosensors for studying interactions between an analyte and immobilised ligand on sensor surface in label-free format; results are obtained in a short time compared to other immunoassays [48,49]. This nanobiosensor’s advantages include rapid analysis time and simplicity when preparing samples since this can be done without having to use markers, developers or other reagents to guarantee suitable reading [50]; exactitude and precision levels are comparable to harmonisation standards for biological sample analysis methods [51,52]. This ensures consistency regarding results and adjusting therapy according to each patient’s conditions and specific needs, including the concomitant use of immunomodulatory therapy without compromising result validity. SPR also enables evaluating affinity and interaction kinetics for a large amount of therapeutic molecules [53,54,55,56], including mAb.

The present study was thus aimed at standardising a label-free real-time methodology for the plasmatic quantification of adalimumab in RA patients who had been treated with such biological therapy.

## 2. Materials and Methods

### 2.1. Materials and Rreagents

The reagents used in this study were a standard active recombinant human TNF-α protein (aa 77-233 aa, ab55237), anti-TNF-α mAb (ab1793) (Abcam PLC, Cambridge, MA, USA), 40 mg/0.8 mL HUMIRA (adalimumab, AbbVie Ltd., Abbott, North Chicago, IL, USA), M1885 ELISA adalimumab level (Sanquin, Amsterdam, The Netherlands). Biacore SPR sensor surface for functionalising assays in the biosensor system, using SCB-CMD200M-5, 10 mM glycine HCl, pH 2.0 (B G20-50), pH 2.4 (B G25-50), pH 3.0 (B G30-50), a desorption kit containing 0.5% sodium dodecyl sulphate (SDS) and 10 mM glycine HCl, pH 9.4 (K D-500). All these reagents were bought from Xan Tec Bioanalytics GmBH (Düsseldorf, Germany). Amino K AN-50 Coupling Kit (containing 1-ethyl-3-(3-dimethylaminopropyl)carbodiimide hydrochloride, 0.05 M N-hydroxysuccinimide (NHS) and 1 M ethanolamine HCl, pH 8.5) (GE Healthcare, Buckinghamshire, UK). HBS-EB buffer (0.01 M HEPES, 0.15 M NaCl, 0.003 M EDTA, 0.005% Tween-20, pH 7.4) was used as running buffer at 5–60 µL/mL flow. Sodium acetate buffer (0.01 M), at 4.0 to 5.5 pH, was used for pre-concentration and immobilisation assays; it was prepared in the laboratory using n 0.22 µm filter and degasified before use. All solutions were prepared with Milli-Q distilled water obtained using the EMD Millipore Direct-Q 3 UV system (Merck KGaA, Darmstadt, Germany) and filtered daily using 0.22 µm Millipore Express Plus system. The anti-TNF-α mAb was used as positive control and HBS-EB as negative control. A reference cell containing 50 µg/mL foetal bovine serum (FBS) (Sigma-Aldrich, St. Louis, MO, USA) at pH 5.0, but without immobilising the solution, was used for suppressing background noise and that from the equipment.

### 2.2. Plasma Ccollection

The study involved recruiting 50 patients and 50 healthy volunteers from an institution offering outpatient rheumatology care (CAYRE-Riesgo de Fractura) in Bogotá, Colombia. The group of patients was selected from a database of people who were being treated with adalimumab. Inclusion criteria involved determining people over 18 years-old having a diagnosis of AR who had been treated with adalimumab for at least 6 months. Exclusion criteria established that pregnant women and patients having had a relapse of base disease during adalimumab treatment were to be excluded. The group of healthy volunteers consisted of people aged over 18 years-old and who had been evaluated by general practitioner/medicine and then submitted to a set of paraclinical tests (haemogram, fasting glucose, TGO, TGP, bilirubin, BUN, creatinine, uroanalysis and BHCG for women). The clinical and paraclinical information was used for verifying each person’s state of health; healthy volunteers’ conditions were determined.

Blood samples (3 mL) were collected in ~1.8 mg EDTA-K tubes for plasma from both groups and in polymer gel tubes for serum determination/separation (Vacutainer, Becton Dickinson & Company, Franklin Lakes, NJ, USA) for healthy volunteers. Samples were handled according to Clinical Laboratory Improvement Amendments guidelines, “necessary for achieving valid results in diagnostic tests” [57]. Each sample was spun and supernatants aliquoted and stored at −20 °C for one week and then deep-frozen at −80 °C until biosensor analysis. The intentions of the study were explained to the groups participating in it; the informed consent forms were read and signed, following Universidad de La Sabana Medicine Faculty Bioethics Committee approval.

### 2.3. Biosensor System

Biosensor analysis was carried out on a Biacore 2000 automatic analyser (GE Healthcare, Uppsala, Sweden), equipped with a microfluidic system and precision liquid injection. The anti-TNF-α quantification strategy was based on direct interaction between TNF-α recombinant protein/anti-TNF-α mAB. Anti-TNF-α detection was based on changes in refraction index produced by ligand (TNF-α protein) and analyte (anti-TNF-α) interaction. As the analyte bound to the ligand these biological entities accumulation of mass it led to an increase in refraction index. Changes in refraction index were measured in real time and the results were graphically represented on a sensorgram, the response being given in resonance units (RU) (arbitrary units) [58]. Plasma samples containing anti-TNF-α were injected into the channel containing the TNF-α recombinant protein ligand by real-time assay format, represented in association/dissociation curves shown in sensorgrams. The instrument and sensor chip were normalised, following the manufacturer’s indications. All experiments were carried out in triplicate at 25 °C with a CMD 200 chip.

### 2.4. Establishing Conditions for Immobilising Recombinant Proteins (Pre-Concentration Assays)

The parameters evaluated in this assay were 0.01 M sodium acetate buffer pH, assay running flow and recombinant protein concentration. Each buffer pH (4.1, 4.5 and 5.0) was explored at three different recombinant protein concentrations (50, 10 and 5 µg/mL) and flow from 5–10 µL/min. A regeneration solution (1 M ethanolamine hydrochloride, pH 8.5) was used between each protein injection as agent for removing protein electrostatically adsorbed on the chip.

### 2.5. Immobilising TNF-α Recombinant Protein

The most critical SPR assay step occurs during immobilisation. Depending on the nature of the ligand (the isoelectric point for proteins), molecular weight, amino acid sequence and other physicochemical characteristics optimum immobilisation conditions on the sensor surface should be found. Once the ligand has been immobilised, epitope availability must be taken into account so that therapeutic antibodies or proteins can be bound [47].

**Figure 1:**
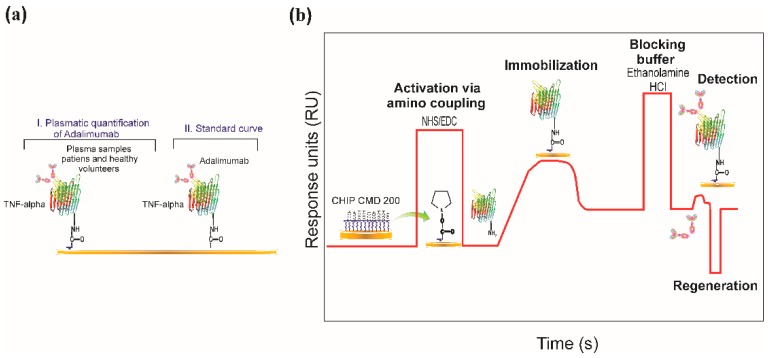
Schematic overview of the direct bioassay. (**a**) Schematic representation of activation via amino coupling on the CMD 200 surface. Carboxyl groups have been activated for covalently immobilising the TNF-α protein. Ab anti-TNF-α is detected once the protein has been immobilised. (I) Plasma samples from patients taking adalimumab and their controls; (II) Procedure for obtaining adalimumab spiked calibration curve in vitro. (**b**) A typical sensorgram obtained by SPR, showing all the stages involved in adalimumab quantification.

TNF-α recombinant protein was immobilised on the CMD200 sensor surface using an amine coupling protocol [59]. Amine-based immobilisation is one of the most used strategies due to its strong covalent binding between sensor surface and a protein and its high sensitivity and orientation specificity [60]. The surface of this chip contains a layer of carboxymethyl-dextran which was activated by an NHS/EDC mixture to produce NHS esters for the reaction containing primary NH_2_-containing ligands. This enabled a covalent link between the ligand and chip surface, once the carboxyl dextran had been activated. The carboxyl groups on the CMD 200 chip surface were activated by exposure to a mixture of equal parts EDC and NHS (1:1) in a 5 µL/min flow for 7 min. TNF-α recombinant protein was diluted in acetate buffer at pH which had been previously selected in pre-concentration assay. Recombinant protein solution was injected onto activated surface until immobilisation level was reached. Excess carboxyl groups remaining activated were blocked with ethanolamine hydrochloride (pH 8.5) at 5 µL/min flow rate for 7 min. Figure 1 gives a typical example of the chemical surface and covalent immobilisation functionalisation steps.

### 2.6. Regeneration Assays

Surface reuse is an additional advantage of using SPR for determining antibodies in plasma or serum compared to other assays. The analyte must be removed but the ligand must remain intact for continuing with new binding assays. Regeneration assays must be used for avoiding complexes forming on sensor surface and analyte-ligand binding results must be reproducible. Strategies reported and used for good regeneration have led to using diluted mixtures of regeneration agents (basics, chelates, non-polar, polar acids and detergents) [61] and strong regeneration solutions [62]. The latter sometimes lead to damage to sensor surface matrix.

Anti-TNF-α regeneration assays were carried out for ensuring complete ligand availability between each injection according to previously reported protocols [61,62] and considering the physicochemical nature of the analyte; 10 mM glycine hydrochloride solutions at 2.0–3.0 pH and 0.00005% SDS were assayed as regeneration agents. The percentage of regenerating analyte obtained for each solution used was calculated using the following equation [1 − (Rreg/Ro) × 100], where Rreg were RU after regeneration solution had been injected and Ro were RU before analyte injection [63,64]. Percentages greater than 80% were considered optimum for continuing analyte injection and ensuring assay consistency and reproducibility. Regeneration solutions were injected in 60 µL volume at 60 µL/min flow rate.

### 2.7. SPR-Based Real-Time Quantification

A standard curve was constructed for quantifying anti-TNF-α mAb by serial dilutions of antibody in HBS-EB buffer with concentration ranging from 1000–20,000 ng/mL. Each antibody dilution was individually injected at 60 µL/min flow rate. The range of linearity, binding capacity, limit of detection (LOD) and limit of quantitation (LOQ) were determined for each assay. LOD was determined by the following equation: (C_LOD_ = 3_(σblank)_/S_conc_) where C_LOD_: analyte concentration σ_blank_: target standard deviation and S_conc_: assay sensitivity (calibration curve slope). LOQ determined by the equation: (C_LOQ_ = 10_(σblank)_/S_conc_). Anti-TNF-α mAb was used as control and was evaluated at 0.01, 0.1 and 1 mg/mL concentrations for determining study specificity. The plasma samples from patients and healthy volunteers were unfrozen at room temperature (RT) and injected in triplicate at 1:100 dilution. Dissociation was monitored for 30 s at 60 µL/min flow rate. Reporting points were established 30 s after finalising each injection. Regeneration followed each measurement by a 30 s pulse of previously chosen regeneration solution at 60 µL/min flow rate for enabling biological and non-biological material on the ligand to be removed. No more than 100 measurements were made per channel, according to SPR protocols. Correlation assay coefficients were calculated for the samples and all biosensor assays were done in triplicate.

### 2.8. ELISA Assays

A commercial ELISA kit reported in the literature (M1885, Sanquim, Plesmanlaan, Amsterdam, The Netherlands) was used for evaluating the groups’ plasma samples and comparing immunoassay results established by SPR; the assays were carried out according to the manufacturer’s indications. A sample of 44 patients and 14 healthy volunteers was chosen for this assay, according to reagent availability. Both groups’ samples were handled in the same way as that for the biosensor assay; all assays were carried in duplicate.

### 2.9. Statistical Analysis

A descriptive analysis was made of the range of adalimumab RU in the groups and the linear correlation between methodologies and ELISA obtained by the assays was estimated by calculating Spearman Rho (ρ) correlation coefficients, given the data distribution where a value of 1 or close to it represented a very high level of correlation of data obtained by both methods. STATA statistical software (version 14.2 for Windows, College Station, TX, USA) was used for statistical analysis of the data. BIA evaluation (version 4.1, GE Healthcare, Uppsala, Sweden) and Origin Lab (version 8.1, Originlab Corporation, Northampton, MA, USA) software was used for analysing the SPR data.

## 3. Results

### 3.1. A Description of the Patients

Information regarding 181 patients being treated with adalimumab was analysed from the revised patient database (i.e., after inclusion and exclusion criteria had been applied); 80 eligible patients were found, of which 50 were finally submitted to clinical and paraclinical evaluation (88% of these patients were female and 12% male). Fifty healthy volunteers were chosen, according to established parameters and paraclinical studies.

### 3.2. Establishing Conditions for Immobilising Rrecombinant Pproteins (Pre-Concentration Assays)

**Figure 2:**
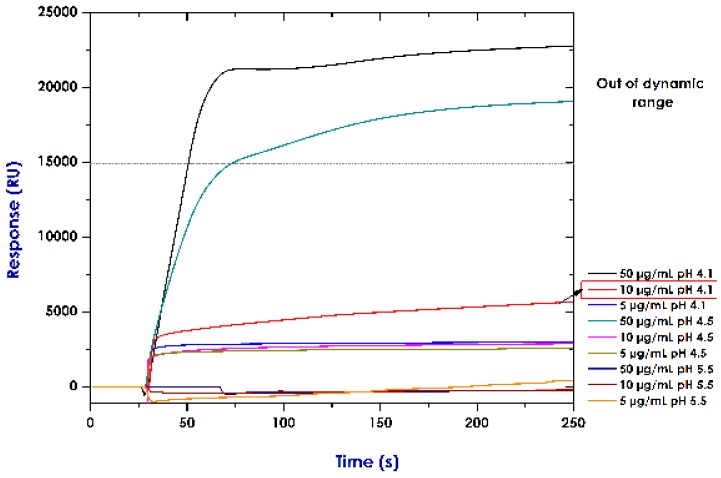
The pH scouting of human recombinant TNF-α (17 KDa molecular mass and 5.8 pI). The protein was diluted in 10 mM sodium acetate buffer, pH ranging from 4.1 to 5.5. Ligand surface concentration on the chip at pH 4.1 was much higher than that obtained at pH 5.5. Optimum pre-immobilisation pH was 4.1 (10 µg/mL).

Figure 2 shows TNF-α recombinant proteins’ electrostatic interactions influenced by pH. The highest protein concentration (50 µg/mL) produced greater adsorption on carboxylated surface: ~15,000 RU relative responses at pH 4.5 and ~22,500 RU at pH 4.1. However, such results were outside the dynamic range established for the equipment. The response (measured in RU) when checking the sensorgram signal and the Biacore 2000 message (signal with a grey bakcground in the sensorgram) were outside the dynamic range.

A dynamic device’s range describes the range of analyte values which can be measured by the post-selected SPR sensing system with specific precision [65]. The regular curves in the sensorgram indicate reflected light intensity compared to incident light angle on sensor surface. SPR is usually used for monitoring changes in reflectance (reflected minimum light) intensity over a range of incident angles when molecules interact on the sensing surface to be then denoted as resonance angles [66]. Thus the meaning of “being outside/beyond the equipment’s dynamic range”, regarding our assays, is that the solution’s refractive index was affected by high protein concentration, producing a very large electrostatic interaction. This, in turn, produced a signal outside the refractive index range established for the equipment, as well as its resonance angles. This would have affected measurement sensitivity.

Relative response was ~5000 RU at 10 µg/mL concentration and 2500 RU at 5 µg/mL at 4.1 pH. Results in RU were very similar for pH 4.5 at 10 µg/mL (~2500 RU) and 5 µg/mL concentrations (2480 RU). The weakest interaction was observed at pH 5.5 for the three established concentrations. This enabled observing the protein concentration’s influence on interaction occurring on carboxymethyl dextran surface (maximum effect at 50 µg/mL concentration). The sensorgram curves for this assay thus showed that the optimum condition for immobilising protein was 10 µg/mL at pH 4.1.

### 3.3. Immobilising TNF-α Recombinant Protein

**Figure 3:**
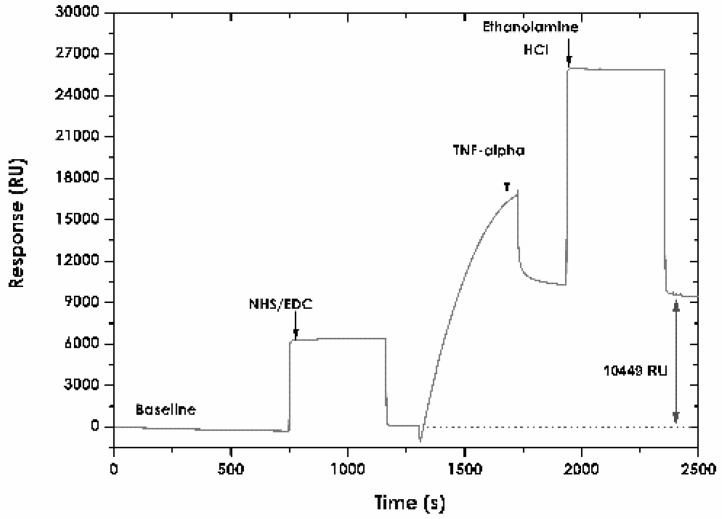
Immobilising TNF-α protein by covalent amine coupling onto the CMD 200 chip. The sensorgram shows TNF-α used as ligand, with its characteristic parts: surface activation (NHS/EDC), ligand attraction and covalent coupling, blocking unoccupied sites (ethanolamine HCl) and final immobilisation response (10,449 RU).

Figure 3 shows the result of immobilising ligand on the CMD 200 sensor surface. The response for immobilising human TNF-α recombinant protein was 10,449 RU. This value was 10.44 ng/mm^2^ surface concentration, based on the equivalence of changing the signal in SPR of 1000 units being equivalent to increasing immobilising matrix mass by about 1 ng/mm^2^ [67].

### 3.4. Regeneration Assays

Regeneration assays must be carried out with suitable chemical solutions to ensure total availability of ligand binding to analyte and good analyte removal. Regeneration was studied using the equation reported in the pertinent literature [58,61,64], ensuring higher than 90% for individual sample injections. Glycine HCl solutions at low pH function very well and are used as agents for optimising regeneration. This may possibly be due to proteins being positively charged at these pHs. Protein binding sites repel each other and this causes molecule remoteness [64]. Other agents such as surfactant solutions (i.e., SDS) enable good removal of analytes and plasma samples. Monitoring base line stability is fundamental for ensuring ligand activity and total removal of remaining bound proteins. The good plasma sample binding assay reproducibility and regeneration percentages ensured specific binding and that the matrix was not damaged.

Sensor surface stability was investigated by making repeated injections of anti-TNF-α mAb and regeneration solutions. Glycine HCl solutions at pH 2.0 were seen to produce very weak regeneration interaction percentages (20%), 2.4 (22.5%) and 3.0 (25%). These pH values for glycine did not produce changes in dissociation curves for reaching initial base line again before injecting the sample. The 0.5% SDS solution produced very strong regeneration, causing an irreversible loss of ligand and thereby its activity; 0.005% SDS solution led to regeneration reaching 70% and 0.0005% led to 90%. SDS at 0.0005% in distilled water was chosen as optimum regeneration solution for the study. Each signal was evaluated in triplicate, using the different regeneration agents.

### 3.5. Calibration Curve

**Figure 4:**
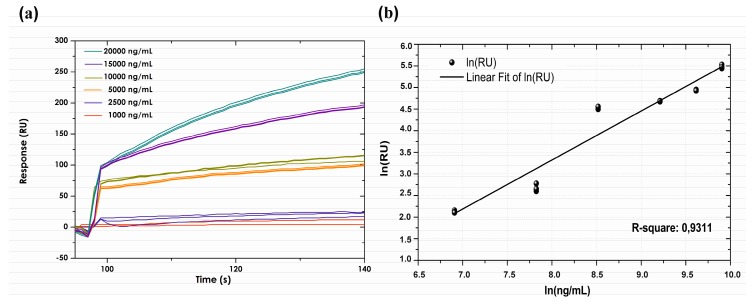
(**a**) Association and dissociation curves for the different adalimumab spiked concentrations in vitro. (**b**) Standard curve for resonance units (RU) compared to anti-human TNF-α. The concentration values used for the standard curve were 1.0, 2.5, 5.0, 10.0, 15.0 and 20.0 µg/mL. R-squared was 0.9311.

A standard curve was constructed from serial dilutions of anti-TNF-α-adalimumab mAb (1.0, 2.5, 5.0, 10.0, 15.0 and 20.0 µg/mL) in HBS-EB buffer. Figure 4 shows this curve; it had a polynomial ratio regarding its relative response. LOD value (defined as the lowest analyte concentration distinguishable in a sample which could be detected but no necessarily quantified as an exact value) was calculated based on response standard deviation, the slope of the curve and equation for straight-line fit of standard points. LOD concentration was estimated at 0.093 µg/mL, whilst LOQ concentration (explained as lower limit for precise measurements) was estimated as being 0.13 µg/mL. Data expressed in RU for each concentration injected was analysed and linearised with high precision (R^2^ > 0.90). The coefficients of variation produced by repeated injections of anti-TNF-α for recombinant protein ranged from 0.001% to 0.011%, finding good assay reproducibility.

### 3.6. Assay Specificity

**Figure 5:**
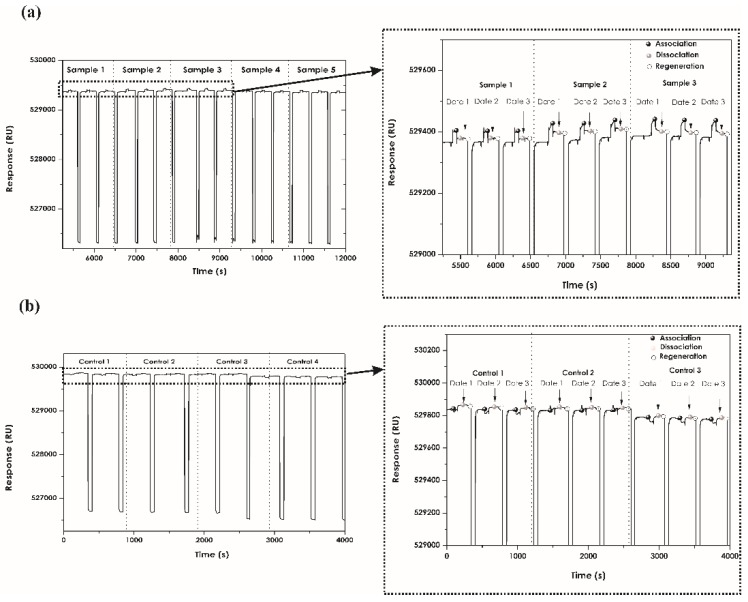
Association and dissociation sensorgrams for replicas of patient (**a**) and control (**b**) samples. Regeneration level with 0.0005% SDS. The RU obtained for patients having anti-TNF-α plasma levels was greater than that for controls (without anti-TNF-α).

When determining assay specificity, it was found that plasma samples from patients treated with adalimumab had association and dissociation interactions reflected in each sensorgram when interacting with recombinant protein immobilised on the chip. There was high repeatability between each injection for the same sample (Figure 5).

**Figure 6:**
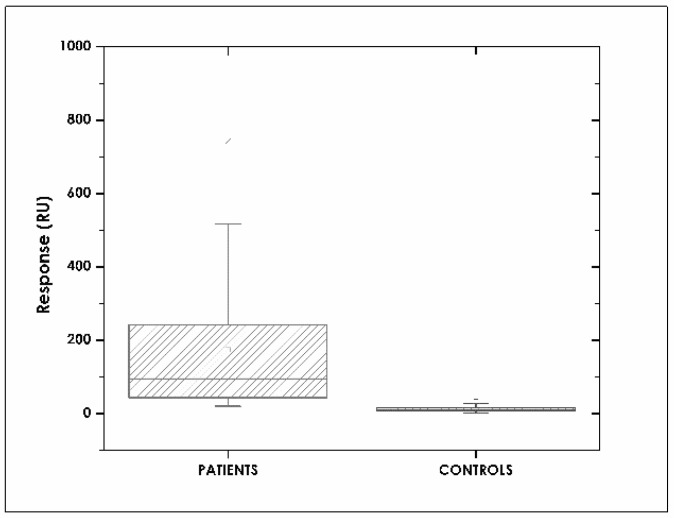
Resonance units (RU) obtained for samples from patients and healthy volunteers; the results were significantly different (*p* < 0.005).

Figure 6 shows the range of adalimumab resonance units (RU) for the patient group, ranging from ~50–250 RU. Injecting plasma samples onto deactivated surface (reference channel) provoked no interaction. These results showed that the chip designed for the interaction was highly specific for anti-TNF-α detection. Healthy volunteers’ samples had an interaction, but values only ranged from ~10 to 20 UR. These values were significantly different to those for the patient group. Plasma samples were diluted 1:100 with HBS-EB buffer.

### 3.7. Correlation between SPR and ELISA Assays

**Figure A1:**
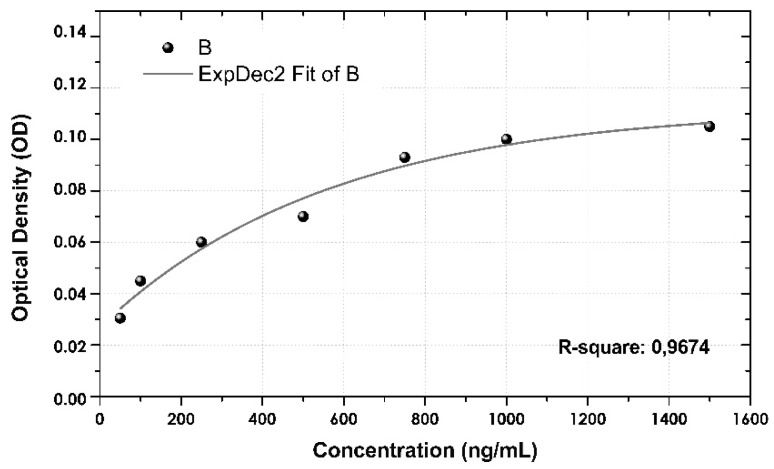
Calibration curve obtained using a commercial ELISA kit.

Bearing SPR immunoassay results in mind, an assay was developed for comparing its methodology to that for ELISA. The commercial kit’s indications were followed to the letter for plasmatic quantification of both study populations. The 1:100 dilution was sufficient for detecting anti-TNF-α concentrations within the calibration curve range (1500–50 ng/mL) constructed for ELISA. Figure A1 gives the ELISA calibration curve.

**Figure 7:**
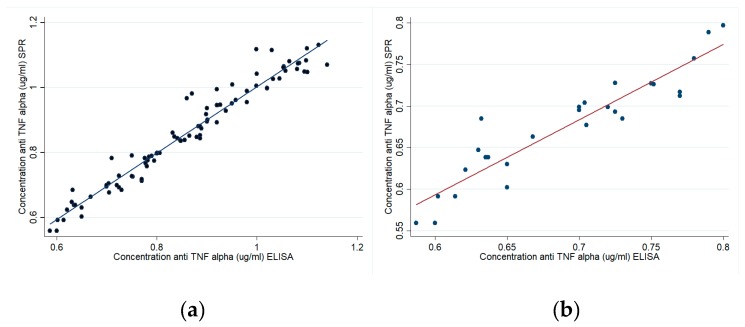
Correlating SPR and ELISA concentrations. (**a**) Patients (R^2^ = 0.97) and (**b**) healthy volunteers (R^2^ = 0.91) sample.

Figure 7a,b shows a 0.97 R^2^ correlation for patients and 0.91 for healthy volunteers regarding response values obtained by SPR and traditional ELISA methodology. Such results suggested that SPR had similar sensitivity to a commercial ELISA kit in quantifying anti-TNF-α in plasma samples.

## 4. Discussion

The SPR strategy used in this study is a direct assay unlike the strategy involved in using commercial ELISA kits (bridging or indirect) [40,68,69]. The commercial ELISA kits used here for evaluating these types of biological drugs were mostly bridging, thereby increasing measurement sensitivity. The bridging strategy has more advantages than direct ELISA since biological fluids’ interference when evaluating anti-TNF drugs and ADA hampers antigen recognition, thereby needing greater sensitivity. However, this strategy is not complete, since functional monovalent antibodies such as IgG4Y cannot be detected on occasions [70].

pH scouting gave values outside the equipment’s dynamic range (50 µg/mL, pH 4.1 and 4.5); it could thus be inferred that the data so obtained at this concentration exceeded the refraction index response limit for Biacore 2000 equipment (i.e., 15,000–30,000 RU, with 1.33–1.36 refraction index) [71]. Because the other 10 μg/mL concentration protein injections at pH 4.1 and 4.5 (shown on the sensorgram) came within the dynamic range (signal shown without grey shading on the sensorgram), it was decided to choose the one having the greatest SPR signal electrostatic interaction. However, refractive indices for solutions at the other two concentrations (10 μg/mL and 5 μg/mL) came within the dynamic range estimated for the equipment. According to reports in the pertinent literature, pre-concentration assays change ligand surface and charge; pH scouting is influenced by ligand concentration and the pH of the buffer used for dilution. Ligand dilution buffer values are usually lower than those for its isoelectric point (pI) (0.5 pH units). High pre-concentration values do not guarantee that a ligand will bind in large quantities [64].

Studies concerned with determining anti-TNF-α using SPR are still scarce. Bian et al., have reported a study evaluating adalimumab in Crohn’s disease patients’ serum by fibre optic SPR (FO-SPR). Anti-TNF-α mAb was immobilised using self-assembling monolayer (SAM) having high detection sensitivity [72,73]. However, (comparing our study to that reported by Bian) we used direct immobilisation which has been widely used in studies involving proteins [17,74], thereby avoiding the SAM formation step and taking advantage of the chip for functionalising it and directly evaluating anti-TNF-α antibodies. The amine coupling-based TNF-α recombinant protein immobilisation in our study thus had good density according to the recommendations for this methodology (+1000 RU). The requirements state that there are no changes in binding activity and/or specificity. The above was demonstrated by the good linearity and sensitivity obtained in the anti-TNF-α mAb calibration curve (R^2^ > 0.90). Another study has reported identifying anti-adalimumab antibodies and characterisation kinetics based on amine route immobilisation, but using an indirect bridging strategy with ELISA [75].

All the parameters were determined for the final part of developing validation assays. Background levels due to non-specific plasmatic protein interactions were monitored in the reference chip and subtracted from sample signal. The *p*-value (<0.0001) confirmed statistically significant differences between study groups. Our results agreed with that reported in the literature stating that plasma and serum samples produce instrumental background noise [75]. However, using a reference channel in SPR-based assays helps in correcting non-specific adsorptions and compensating for refraction index differences [76]. An mAb-anti-TNF-α positive control was used for ensuring that Humira drug excipients did not cause antibody noise in interactions, unlike adalimumab. No interactions were observed which were different to those observed with the drug. Quantitative determination of patients’ samples’ binding levels was possible once the interaction range had been established.

Studies for evaluating adalimumab by Bian et al., showed plasma concentrations ranging from 19 to 0.3 µg/mL (SPR) [75] agreeing with results of the same magnitude reported in other studies which had been determined by evaluating three types of commercial ELISA, values ranging from 20 to 0.5 µg/mL [39,77,78]. Our results for patients’ samples agreed with the previously reported magnitude. The values reported in the literature were 0.2–6 µg/mL for the commercial ELISA kit used in our study [39]. The data from our research agreed with both SPR and ELISA concentration. It is worth noting that the presence of ADA could have made drug concentration detection vary. It would be interesting to carry out more studies on this topic even though this was not the objective of this research. Previous reports regarding SPR-related assay speed have given an estimated 5 min delay from the time of injection until results are obtained (after standardising all conditions) [58], compared to an average 12–16 h when using an ELISA kit [78], thereby highlighting the assay’s ease of use and speed for this type of protein using SPR.

Our study had some limitations. We were only able to evaluate a limited amount of samples by ELISA due to (a lack of) reagent availability. Secondly, ELISA assays were measured in duplicate, just once. Such assays are probably carried out in this way in clinical practice, with concentrations only being determined once.

## 5. Conclusions

An SPR immunoassay-based label-free assay was successfully developed in this study. This methodology could be used in routine clinical practice for TDM regarding biologics and biosimilars. Real-time quantification should enable optimising the costs involved in biological therapies as well as minimising therapeutic failures in patients.

## Appendix A

This section gives the calibration curve obtained for ELISA immunoassays using a commercial kit.
